# Correction: Gajurel et al. Arbovirus in Solid Organ Transplants: A Narrative Review of the Literature. *Viruses* 2024, *16*, 1778

**DOI:** 10.3390/v17030292

**Published:** 2025-02-20

**Authors:** Kiran Gajurel, Reshika Dhakal, Stan Deresinski

**Affiliations:** 1 Division of Infectious Diseases, Carolinas Medical Center, Atrium Health, Charlotte, NC 28204, USA; 2 Labcorp, Indianapolis, IN 46214, USA; reshika.dhakal@labcorp.com; 3 Division of Infectious Diseases and Geographic Medicine, Stanford University, Stanford, CA 94305, USA; polishmd@stanford.edu

## Error in Table

In the original publication, there was a mistake in Table 1 as published [1]. Mayaro virus was mistakenly listed under *Flaviviridae* family. It should have been listed under ‘*Togaviridae* family’. Also, under *Bunyaviridae* family (Bunyavirales), “Rift Vallet fever” virus should be corrected to “Rift Valley fever virus”.

## Figures and Tables

**Table 1:** Medically significant arboviruses.

***Togaviridae*** **family:**
Eastern equine encephalitis virus
Western equine encephalitis virus
Venezuelan equine encephalitis virus
Chikungunya virus
Sindbis virus
Ross river virus
Barmah Forest virus
Mayaro virus
***Flaviviridae*** **family:**
Dengue virus
Japanese encephalitis virus
Murray valley fever virus
St Louis encephalitis virus
West Nile virus
Powassan virus
Tick Borne encephalitis virus
Louping ill virus
Omsk hemorrhagic fever virus
Alkhurma Hemorrhagic Fever Virus
Kyasanur Forest Disease Virus
Zika virus
Yellow fever virus
Usutu virus
Rocio encephalitis virus
***Bunyaviridae*** **family (Bunyavirales)**
California encephalitis virus
-La Crosse encephalitis virus
-Jamestown Canyon
Rift Valley fever virus
Crimean Congo hemorraghic fever virus
Severe fever with thrombocytopenia syndrome
Heart land virus
Oropouche Virus
Toscana Virus
Cache Valley fever
Ngari Virus
***Reoviridae*** **family**
Coltivirus
-Colorado tick fever virus
-Seadornavirus (Banna virus)
***Orthomyxoviridae*** **family**
Thogotovirus genus including thogoto, bourbon and dhori viruses
