# Supplementary material for: Arbovirus in Solid Organ Transplants: A Narrative Review of the Literature
Source: Viruses. 2024 Nov 15;16(11):1778. doi: 10.3390/v16111778 (PMC11599096; doi:10.3390/v16111778)
Supplement: Supplementary file 1 [file viruses-16-01778-s001.zip › viruses-3260246-supplementary.pdf]

Supplementary material S1: Methods used in selecting articles for the review

Published literature limited to the English language on arbovirus and transplant was searched in Pubmed through July 13, 2024 using search terms 'transplant' or 'transplantation' combined with 'arbovirus' and the following individual viruses (The viruses were searched with and without using the term 'virus'): eastern equine encephalitis , western equine encephalitis, venezuelan equine encephalitis , chikungunya, sindbis, ross river, barmah forrest virus, dengue, Japanese encephalitis, murray valley fever, St Louis encephalitis, west nile , powassan, tick borne encephalitis, louping ill virus, Omsk hemorrhagic fever, alkhurma hemorrhagic fever, Mayaro, kyasanur forest disease, zika, yellow fever, usutu, rocio encephalitis, California encephalitis, La crosse encephalitis , Jamestown canyon, rift valley fever, crimean congo hemorraghic fever, severe febrile thrombocytopenia syndrome, heartland virus, oropouche , toscana, ngari, cache valley, coltivirus, Colorado tick fever, seadornavirus, banna virus, thogoto, bourbon and dhori . In addition, relevant references from the retrieved articles were also reviewed.

Only articles that reported arbovirus infection in solid organ transplant (SOT) were selected for the purpose of the review. We excluded articles that reported infection in cornea transplant only. The following arbovirus infections were found to be associated

with SOT and are reviewed in the paper: dengue, Japanese encephalitis, chikungunya, yellow fever, zika, powassan , tick borne encephalitis, West Nile, eastern equine encephalitis, St Louis encephalitis, Jamestown canyon, cache valley, Crimean-Congo hemorrhagic virus, heartland virus, rift valley fever and usutu virus.
